# A qualitative systematic review of the social eating and drinking experiences of patients following treatment for head and neck cancer

**DOI:** 10.1007/s00520-021-06062-7

**Published:** 2021-03-01

**Authors:** Mark Dornan, Cherith Semple, Anne Moorhead, Eilís McCaughan

**Affiliations:** 1 School of Nursing, Institute of Nursing and Health Research, Ulster University, Newtownabbey, UK; 2 Cancer Services and Ulster Hospital, South Eastern Health and Social Care Trust, Belfast, UK; 3 School of Communication and Media, Institute of Nursing and Health Research, Ulster University, Newtownabbey, UK; 4 School of Nursing, Institute of Nursing and Health Research, Ulster University, Coleraine, UK

**Keywords:** Head and neck cancer, Cancer survivorship, Eating, Social, Systematic review

## Abstract

**Purpose:**

Patients living with and beyond head and neck cancer (HNC) often have long-term, functional challenges as a result of treatment. A key functional challenge relates to eating and drinking; often associated with physical, emotional, and social difficulties. Eating and drinking with family members and friends can become a struggle, increasing the risk of social isolation and loneliness. This systematic review aims to identify and synthesise the literature on the experiences of social eating and drinking for patients following treatment for HNC.

**Methods:**

Six electronic databases (Pubmed, Web of Science, CINAHL, EMBASE, PsychINFO, and Scopus) were systematically searched using subject headings and free-text word searches in February 2020. Citation chaining and Google Scholar were used to identify grey literature. PRISMA procedures were followed.

**Results:**

Of 6910 records identified, 24 studies met the inclusion criteria. Synthesis of the research findings results in two major themes: (1) the experience of loss associated with social eating and drinking, and (2) adjusting and support to promote social eating and drinking.

**Conclusion:**

Losses associated with social eating affect a patient’s psychological and emotional well-being and impact on close relationships. To promote positive participation in social eating, patients were more likely to seek and receive support from someone within their close social network, rather than a healthcare professional. Family and friends are an essential source of support and are integral in facilitating engagement with social eating following treatment for HNC. Future interventions should promote family orientated resources, incorporating self-management strategies.

**Supplementary Information:**

The online version contains supplementary material available at 10.1007/s00520-021-06062-7.

## Introduction

The concept of social eating and drinking is to eat or drink in the presence of another person [1]. Eating and drinking socially is also known as commensality, which in a literal sense means to come together at a table [2, 3]. Social eating may take place as a daily activity, with most people eating at least one or two meals with another person each day, primarily, those with whom they live [4, 5]. Eating with colleagues or going to cafés and restaurants to meet friends has become a regular aspect of modern life and an opportunity to bring people together. Social eating and drinking are observed as integral aspects of cultural, religious, and celebratory occasions [6–8].

Literatures illustrate that eating and drinking have a central and significant meaning to peoples’ lives [6]. Eating and drinking are observed and enjoyed as more than a physical activity, and its meaning extends beyond the value of nutrition [9]. Sharing meals with others provides the opportunity to engage in everyday casual conversation and to share experiences [6]. Eating socially nurtures relationships and is more likely to make people feel better about themselves, participate in a broader social network and obtain emotional support [4].

For patients with head and neck cancer (HNC), eating and drinking with others are reported as a significant challenge [10]. Research reports that up to 90% of patients with HNC have eating and drinking difficulties after treatment [11, 12]. A potential range of side effects can inhibit a patient’s ability to eat and drink, including pain, xerostomia, mucositis, nausea, lack of appetite, dysphagia, and dysgeusia [11, 13].

A growing body of evidence indicates the challenges of social eating and drinking for patients with HNC [14, 15]. A literature review by Ganzer et al. [14] on the changed meaning of food identified the importance of the social dimension of food and drink for patients with HNC. Patients with HNC are at potential risk of social isolation, loneliness, and reduced quality of life from the functional eating and drinking difficulties encountered as a result of treatment [15]. There have been no systematic searches or literature syntheses on the experiences of social eating and drinking for patients with HNC. This is a fundamental gap in our current understanding of the survivorship experiences of patients living with and beyond HNC.

A systematic synthesis of the literature will establish opportunities to raise healthcare professionals’ (HCP) awareness and inform them of the long-term social eating and drinking adversities encountered by patients with HNC. Furthermore, this review will help identify and consolidate the key areas of support and inform the planning, development, and delivery of evidence-based support to address these challenges. Therefore, the aim of this review is to identify and synthesise the experiences of social eating and drinking of patients living with and beyond HNC. The objectives of this systematic review are:

- To explore the social experiences of eating and drinking of patients following treatment for HNC.
- To identify the support needs surrounding social eating and drinking for patients following treatment for HNC.
- To identify strategies to promote social eating and drinking for patients following treatment for HNC.

## Methods

The systematic review followed an a priori protocol adhering to the Preferred Reporting Items for Systematic Reviews and Meta-analyses (PRISMA) [16] (Electronic Supplementary Material 1). The systematic review protocol was registered on with the International Prospective Register of Systematic Reviews (PROSPERO) with registration number CRD42020162875.

### Search strategy

Six electronic databases were used to identify relevant literature: Pubmed, Web of Science, Cumulative Index of Nursing and Health (CINAHL), Excerpta Medica Database (EMBASE), PsycINFO, and Scopus. The search terms were developed from the key concepts of the review aim: ‘head and neck cancer’, ‘eating and drinking challenges’, and ‘experiences’. Free-text word searches and subject heading searches were used as appropriate to each database to ensure a comprehensive search. The final list of terms was clarified with an experienced librarian, confirming a systematic approach was undertaken between different databases. Grey literature was reviewed from Google Scholar to ensure complete coverage. Citation chaining was used to identify eligible works from the references of the included studies. ‘Wildcards’ were also used to search for partial words, alternative spellings, and pluralisation. All searches were completed on 17 February 2020. The database searches are displayed in Electronic Supplementary Material 2. All results were collated in RefWorks.

### Study eligibility

Study inclusion criteria consisted of (1) patients aged 18 years and over who had completed treatment for HNC, (2) research described a patient’s experience of social eating and drinking, (3) the publication was primary research. Studies were excluded as follows: (1) secondary research (e.g. reviews, opinion articles, editorials), (2) papers reporting on the physical and functional impact of HNC only without reporting the social experiences of eating and drinking relating to HNC, (3) studies that report on more than one type of cancer, where findings cannot be separated to results of an HNC cancer group. Retrieved studies were published in English from January 2009 to December 2019 to obtain current evidence on this survivorship challenge for patients with HNC.

### Screening

**Fig. 1:**
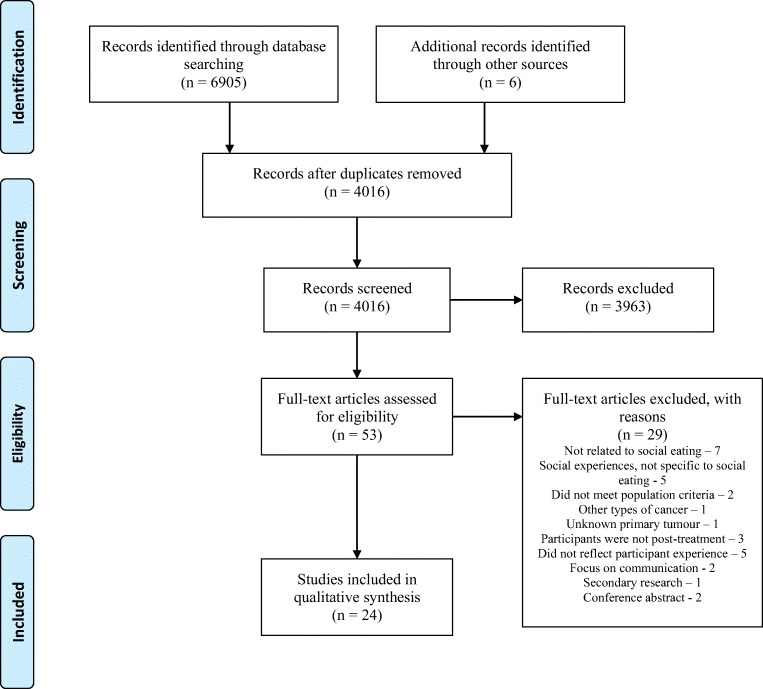
PRISMA flowchart

Duplicates were removed. Titles and abstracts were reviewed using the eligibility criteria by one reviewer (MD). This process was checked by a second reviewer (CS). Full-text papers were obtained for studies meeting the eligibility criteria or if the eligibility could not be determined from the title and abstract screen. Two authors were contacted for clarification of their work. Full-text papers were screened using a screening tool developed by the research team to assess eligibility and to ensure rigour (Electronic Supplementary Material 3). Included papers were verified by CS, and a decision on indeterminate studies was reached by discussion with CS. This process is displayed in Fig. 1.

### Data extraction and synthesis

The following characteristics were extracted from the included studies by the first author (MD): author(s), year, country, study aim(s), research design, data collection methods, sample number and characteristics, and key finding(s), displayed in Table 1. Relevant findings from each paper were extracted verbatim into a spreadsheet. Research findings were synthesised using *Thomas and Harden’s* [41] three-step approach to thematic analysis. Initial codes were generated in the first instance by using a word or phrase to describe the finding. This was completed in an iterative process. These codes were then grouped into descriptive themes using an inductive approach as there were no predetermined categories in which data should be placed. Finally, the descriptive codes were developed into analytical themes through a cyclical process and discussion. The final analytical themes were refined by the research team (MD, CS, AM, EM) and checked by rereading each paper to ensure they reflected the meaning and essence of the literature.

**Table 1:** Data extraction table

Author(s), year and country	Study aim	Research design	Data collection	Sample characteristics	Main study findings
Alberda et al. (2017) [17] Canada	To explore patients’ perspectives on nutrition care in the context of their illness, medical treatment, and recovery	Qualitative	Semi-structured interview	20 participants, 10 with oesophageal cancer (8 male and 2 female) 10 with HNC (8 male and 2 female). Age range 45–79 years. Treatment: surgery only—1; RT only—1; surgery/RT/CT—12; surgery/RT—6.	(1) Coping with physical and psychosocial aspects of illness and nutrition; (2) understanding the nature of the illness, treatment, and nutrition pathway; and (3) being supported during the trajectory of care.
Burges-Watson et al. (2018) [18] England, UK	To create a multi-dimensional framework to facilitate systematic assessment and development of a comprehensive intervention	Qualitative	Video-reflexive ethnography	25 participants with HNC (14 male and 11 female) and partners. Age range 54–65. Treatment: (chemo)radiotherapy—25.	The development of an altered eating framework to assess a patient’s relationship with food over 7 domains after treatment for HNC.
Checklin et al. (2019) [19] Australia	To investigate patients’ perspectives on their experience of oropharyngeal dysphagia rehabilitation after treatment for HNC	Qualitative	Semi-structured in-depth interview	8 participants with HNC (6 male and 2 female). Age range 51–75 years. Treatment: surgery only—7; surgery and RT—1.	(1) The supportive network is essential; (2) reassurance from staff professionalism; (3) access to service; (4) using own motivation and resilience; (5) receiving the right information; (6) need for future research.
Dooks et al. (2012) [20] Canada	To describe the experience of community reintegration following laryngectomy surgery	Qualitative	In-depth interview	9 participants who had total laryngectomy surgery (8 male and 1 female). Age range 60–75 years. Treatment: total laryngectomy surgery and RT—9.	There was constant accommodation to life with a laryngectomy. Three main themes: (1) impact of cancer diagnosis; (2) coping with illness; and (3) transitions to recovery.
Dunne et al. (2019) [21] Ireland	To describe the ways in which HNC survivors begin to integrate self-management into their daily lives	Qualitative	Semi-structured interview	27 participants with HNC (18 male and 9 female). Age range 25–70+ years. Treatment: surgery and RT—10; surgery, RT and CT—11; RT and CT—6.	(1) Grappling with self-management; (2) trying different strategies; (3) becoming an expert; (4) struggles; (5) avoiding recommendations; and (6) interpreting self-management.
Einarsson et al. (2019) [22] Sweden	To describe patients’ experiences of food and eating 2 years after treatment and how they cope	Qualitative	Thematically structured interview	135 patients with HNC (100 male and 35 female). Age range 34–87 years. Treatment: RT—49; surgery—4; RT then surgery—34; surgery then RT—29; CT, RT, and/or surgery—19.	(1) The constant battle; (2) food alterations and nutritional support; (3) not joining in; (4) coping; (5) relationships; and (6) longing for ‘normality’.
Ganzer et al. (2015) [23] USA	To explore the eating experience of survivors of HNC up to 3 years after chemoradiation	Mixed methods	Interview and Vanderbilt Head and Neck Symptom Survey 2.0	10 patients with HNC (7 male and 3 female). Age range 40–67 years. Treatment: induction CT—7; concurrent chemoradiation (CCR) therapy only—1; CCR and surgery—2; induction CT, CCR, surgery—3; concurrent chemotherapy—10.	(1) The psychological impact; (2) functional impact; (3) social impact; and (4) eating experience. These were encompassed by the overarching need to adapt.
Goswami and Gupta (2019) [24] India	To understand the problems faced by patients with oral cancer from diagnosis until end of treatment	Qualitative	In-depth interview	24 patients with HNC (18 male and 6 female). Age range 35–82. Treatment: Surgery and RT—8; surgery, RT, and CT—7; surgery, RT, and PL—2; RT, CT, and PL—2; surgery, RT, CT, and PL—5.	Post treatment challenges included: (1) concerns for quality of life; (2) social constraints; (3) financial security; and (4) feeding problem.
Jiang et al. (2017) [25] China	To describe the experience of radiation-induced xerostomia in the daily lives of Chinese patients with HNC	Qualitative	Semi-structured interview	20 patients with HNC (13 male and 7 female). Age range 29–80 years. Treatment: RT and adjuvant CT—6; RT only—5; concurrent CT—5; RT and surgery—2; surgery, CT, and RT—2.	Five categories identified in relation to xerostomia from HNC treatment: (1) communication problems; (2) physical problems; (3) psychosocial problems; (4) treatment problems; and (5) relief strategies.
McQuestion et al. (2011) [26] Canada	To explore the experiences of patients’ receiving radiotherapy and the disruptions caused by treatment	Qualitative	Interview	17 patients with HNC (12 male and 5 female). Age range 30–70+ years. Treatment: Daily RT—17; BID radiation—5.	The meaning of food had changed and was evident in three aspects of people’s lives: (1) physical; (2) emotional; (3) social.
Molassiotis and Rogers (2012) [27] England	To explore experiences, over a 1-year period, of issues and concerns described by patients with HNC	Qualitative	Semi-structured interview	16 patients at T1 (14 male and 2 female), 13 at T2, 12 at T3, and 10 at T4 with HNC. Age range 34–80 years. Treatment: RT—8; CT and RT—4; surgery—4.	Four prominent issues reported up to 1 year by patients: (1) nutritional concerns; (2) tiredness; (3) the radiotherapy mask; (4) regaining ‘normality’.
Moore et al. (2014) [28] Australia	To explore the experiences of patients who received treatment for HNC, describe support needs and managing unmet needs	Qualitative	Semi-structured interview	8 patients with HNC (7 male and 1 female). Age range 51–60 years. Treatment: CT—3; surgery and RT—2; surgery, RT, and CT—3.	Findings were organised using the stress, appraisal, and coping model and describe the areas for support and the negative impact on quality of life post-treatment.
Mortensen and Paaske (2012) [29] Denmark	To explore the long-term quality of life of people who have tonsil cancer	Qualitative	Semi-structured interview	7 patients with tonsil cancer (3 male and 4 female). Age range 54–65 years. Treatment: RT and surgery—4, RT, CT, and surgery—3.	The side effect of treatment was greatest at 3 months after treatment. People reported impact on QoL even 2 years after treatment.
Nund et al. (A) (2014) [30] Australia	To explore the lived experience of the impact of dysphagia following HNC management	Qualitative	Interview	24 patients with HNC (20 male and 4 female). Age range 43–71 years. Treatment: RT + systemic therapy—23; RT only—1.	Four main themes of the experience of dysphagia following treatment: (1) physical changes; (2) emotions response; (3) altered meaning of food; (4) personal and lifestyle impacts.
Nund et al. (B) (2014) [31] Australia	To explore the experience of dysphagia following non-surgical treatment for HNC the perceptions of service needs	Qualitative	Interview	24 patients with HNC (20 male and 4 female). Age range 43–71 years. Treatment: RT + systemic therapy—23; RT only—1.	There are five interrelated themes to this study: (1) life after treatment; (2) practical adjustments living with dysphagia; (3) emotional adjustments; (4) accessing support outside the hospital; and (5) perceptions of dysphagia-related services.
O’Brien et al. (2012) [32] Ireland	To explore the experiences of change within intimate relationships due to HNC	Qualitative	Semi-structured interview	16 patients with HNC (12 male and 4 female). Age range 35–71. Treatment: surgery only—5; surgery + RT—8; surgery + chemo-radiotherapy—1; chemo-radiotherapy—2.	Three major themes demonstrate the changes in intimacy of relationships following treatment: (1) personal identity; (2) re-establishing social networks; and (3) intimate relationships.
Ottosson et al. (2013) [33] Sweden	To describe the experience of food, eating, and meals after radiotherapy treatment for HNC	Qualitative	Interview	13 patients with HNC (11 male and 2 female). Age range 47–70 years. Treatment: RT only—6; RT + surgery—7.	Findings suggest six post-treatment categories of patients’ experience: (1) a long journey; (2) a new way of eating; (3) eating without satisfaction; (4) challenging meals outside the family; (5) support and information; and (6) a new normal.
Parahoo et al. (2019) [34] Northern Ireland	To explore the experience of dental loss in patients with HNC	Qualitative	Semi-structured interview	15 patients with HNC (10 male and 5 female). Age range 51–80 years. Treatment: RT—2; RT + CT—3; surgery + RT—7; surgery + RT + CT—2; surgery only—1.	Post-treatment experiences include (1) impact of dental loss; (2) coping with dental loss; and (3) getting dentures and implants.
Pateman et al. (2015) [35] Australia	To describe how people with HNC cope with altered oral function and to identify their supportive care needs	Qualitative	Semi-structured interview	6 patients with HNC (4 male and 2 female). Age range 50–72 years. Treatment: RT + CT—3; surgery—1; surgery + RT—2.	Three key themes describing patient experiences of altered oral function: (1) dimensions of eating; (2) maintaining oral health; and (3) adapting to the chronic side effects.
Patterson et al. (2015) [36] England	To describe HNC patients’ experiences of change of swallowing challenges following chemoradiotherapy	Qualitative	12 patient observations, 4 with partner present. 6 individual interviews and 3 dyad interviews.	Phase 1: 12 patients with HNC (10 male and 2 female) and 4 partners. Age range 45–77. Treatment: CT—10; RT—2. Phase 2: 9 patients with HNC (8 male and 1 female) and 3 partners. Age range 50–72 years. Treatment: CT—7; RT—2.	Findings include early post-treatment and late post-treatment experiences. Eating and drinking issues are highly individualised and have pervasive physical, social, and practical aspects.
Semple et al. (2019) [37] Northern Ireland	To explore the long-term impact of living with an obturator to rehabilitate a maxillary defect	Qualitative	Semi-structured interview	12 patients with HNC (8 male and 4 female). Age range 38–84. Treatment: surgery only—7; surgery + RT—4; surgery + RT + CT—1.	The experience of living with an obturator is demonstrated across (1) preparedness for living with an obturator; (2) impact of living with an obturator; (3) stability and retention of an obturator; and (4) coping strategies.
Sterba et al. (2017) [38] USA	To characterise primary end-of-treatment challenges in HNC to assist the development of a survivorship needs assessment planning tool	Qualitative	Semi-structured interview	17 patients with HNC (10 male and 7 female). Age range 33–75 years. Treatment: surgery—82%; CT—59%; RT—82%. 14 caregivers (6 male and 8 female). Age range 29–83.	The findings highlighted the post-treatment physical, emotional, and social challenges and a wide variety of complex follow-up care experiences and testing of the Survivorship Needs Assessment Planning (SNAP) tool.
Tong et al. (2011) [39] Hong Kong	To gain patients’ perspectives and experiences of post-irradiation swallowing difficulties	Mixed-methods	Semi-structured in-depth interview and self-report questions	60 with nasopharyngeal cancer (42 male and 18 female). Age range 34–71 years. Treatment: RT—60.	Post-irradiation experiences include (1) patient judgement of swallowing difficulties; (2) definitions of a normal diet; (3) the perceptions of ‘no difficulties’; and (4) little attention paid to dysphagia symptoms.
Zou et al. (2015) [40] China	To understand how treatment for tongue cancer affects daily life at 1 year following glossectomy with free flap reconstruction	Qualitative	Semi-structured interview	16 male patients with tongue cancer. Age range 34–64 years. Treatment: partial glossectomy and free thigh flap reconstruction—16.	Patients described physical, social, relational, and emotional changes, change to sexual practice, and use of traditional Chinese medicine.

*RT*, radiotherapy; *CT*, chemotherapy; *PL*, palliative therapy

### Quality assessment

Each paper was quality assessed using the Critical Appraisal Skills Programme (CASP) [42] tool by MD and verified by CS. The CASP tool is a commonly used method to appraise studies in qualitative synthesis and consider research transparency and methodological appropriateness [43]. Each response in the CASP tool was assigned a numerical value (Yes=1, Can’t tell=0, No=0). A total score was then calculated for each included study with a maximum possible score of 9. A summary table is included in Electronic Supplementary Material 4. Any differences of opinion were resolved by discussion.

## Results

The search identified 6905 records (Pubmed (*n*=1091), Scopus (*n*=1437), PsycINFO (*n*=57), CINAHL (*n*=631), Web of Science (*n*=1373), EMBASE (*n*=2316)) with 4015 remaining after the removal of duplicates. In total, 53 titles met the eligibility criteria to have a full-text screen. Subsequently, and after a further screening, 25 articles were excluded. Four further papers were later discussed with CS and on agreement, subsequently eliminated from the final inclusion as they did not meet the eligibility criteria. The final total number of papers included was 24. A member of the research team verified each included study. A record of the decision to include and exclude papers with reason was kept. Reasons for exclusion are included in Fig. 1. All the included studies reported findings on patients’ experiences of social eating and drinking after treatment for HNC.

### Participants

Within the 24 included studies, a heterogenous HNC population of 516 patients (male=379, female=137) was identified. Sample sizes ranged from 6 to 135. These included participants diagnosed across a range of tumour locations such as oral cavity, tonsil, larynx, and pharynx, of various stages and receiving different modalities of treatment. Twenty-one studies included patients from different HNC subsites. Three papers focused on patients with one type of HNC: oral cancer [24], tonsil cancer [29], tongue cancer [40]. Within the studies, the treatment a person received resulted in unique physical and functional effects, both in the acute and long-term recovery that impacted social eating and drinking. Some patients living with an obturator following a maxillectomy experienced nasal leakage and altered chewing [37], whereas dental loss often contributed to issues with biting, swallowing, and talking [34]. Partial glossectomy frequently led to limited tongue mobility [40], and a common side effect following radiotherapy was xerostomia [32]. Other functional challenges that directly impacted on HNC patients’ ability to participate in social eating and drinking activities were coughing [20, 22, 33], noise from eating and swallowing [28], swallowing difficulties [30, 35, 39], and oral incontinence [20, 37]. The physical and functional challenges people encountered with eating and drinking led to an altered eating experience [18].

### Results of synthesis

Two themes were developed to illustrate the reported social eating and drinking experiences for patients with HNC: (1) the experience of loss associated with social eating and drinking and (2) adjusting and support to promote social eating and drinking.

#### Theme 1: The experience of loss associated with social eating and drinking

Physical alterations for patients with HNC had significant repercussions on their ability, perceived ability, and confidence to engage in social activities involving food and eating. Patients regarded their experience of social eating as a loss. The experience of loss is depicted across three subthemes: (1) loss of ability and confidence to eat in a socially acceptable way, (2) loss of social participation and enjoyment associated with normal eating and drinking, and (3) loss of togetherness with family and friends. For the context of this review, social eating refers to both eating and drinking.

##### Theme 1.1: Loss of ability and confidence to eat and drink in a socially acceptable way

The effects of HNC and the impact of treatment regularly prevented people from sharing meals in a social situation [25, 26, 35]. Often, patients perceived they had lost the ability to eat and drink with others in a socially acceptable way. Patients felt embarrassment, shame, and were self-conscious about not being able to control their physical symptoms, such as drooling and nasal leakage; highlighting how this led to ‘making a mess’ in social situations [18, 21, 22, 28, 33, 35–37]. Selecting food to eat became a conscientious process, and patients felt unable to eat like a ‘normal’ person [22, 24, 30]. Occasionally patients became anxious when they were invited to someone’s house. They had a fear of insulting the host if they were unable to eat the food being served but also did not want attention drawn to the situation, with a ‘fuss’ being made over not being able to eat or drink [25, 33, 36].

##### Theme 1.2: Experiencing loss of participation and enjoyment associated with social eating and drinking

Participants reported eating less regularly with others after their treatment and for some, this was such a challenge that they excluded themselves from social occasions and chose to remain at home [21, 25, 26]. Meeting with friends and family in restaurants or cafés became a less common occurrence [22, 28, 36]. Patients reported that they refrained from attending special celebrations, such as weddings [34, 36, 37], Christmas [37], and New Year parties [25] due to the functional and psychological challenges of eating and drinking. This sometimes extended to the type of holidays people were able to take [30]. Not participating in social eating activities restricted patient’s social lives and often isolated them from others [35, 36].

Some patients, however, who attended social eating events appeared to not have the same sense of enjoyment as before treatment and believed that they no longer made the same contribution to the social environment [22, 27, 40]. For example, residual impairment meant that people were unable to talk and eat at the same time, which made engaging in mealtime conversation more arduous [22–27]. Additionally, the noisy surroundings in restaurants were not conducive to conversation and therefore required more effort to participate [19, 20]. The uncertainty of the availability of suitable food resulted in diminished enjoyment when eating out, and participants regarded this as a loss [20, 32, 39]. The loss of social participation was not solely limited to going out of the house to eat and drink but also impacted mealtime participation at home.

##### Theme 1.3: Experiencing loss of togetherness with family and friends

The findings demonstrated that following HNC treatment, patients shared meals less frequently with family and friends, which had an impact on relationships. Regular meals at home were no longer shared as a family, and patients ate separately as family members felt guilty or uncomfortable enjoying a meal that the person could not share [18, 30, 38]. Consequently, patients experienced a loss of togetherness with their friends and family [30]. Family members became irritated at the length of time it took to eat a meal, which affected their relationship [39]. The process of eating often required more time which could result in the patient remaining at the table alone to finish their meal and consequently feeling more isolated [28, 33, 39]. Some patients indicated that they no longer received invitations to social events from friends or family members, as eating would be involved [23]. For this reason, patients reported to be less motivated to socialise with others [28]. The loss of togetherness extended from friends, family, spanning to work colleagues [37].

#### Theme 2: Adjusting and coping to promote social eating

Despite the challenges and the associated losses caused by HNC treatment, over time, many patients found methods of adjusting and adapting to enable coping with social eating [40]. Some of the coping strategies demonstrated by patients, such as avoidance and isolation limited their participation in social eating opportunities [25, 37]; however, alternative methods of adapting facilitated positive participation with eating socially. By adopting adjustment strategies, some people were able to reduce social embarrassment and disruptions to their social eating [40]. This was demonstrated by the two themes: (1) taking control of the social eating situation and (2) engaging with support.

##### Theme 2.1: Taking control of the social eating situation

Being candid about their cancer and describing how the disease affected their eating and drinking helped demonstrated ownership over their situation [22]. When going to restaurants, some patients would ask for discrete places to sit to be able to eat more privately and feel less conspicuous [22, 36, 37]. Others contacted restaurants in advance to ensure there would be food that they would be able to eat [35]. This extended to going on holidays where they could ensure food preparation met their individual needs. Cruises appeared to be a good option [30]. Food modification was a crucial aspect of coping with social eating and drinking challenges. Smaller, more comfortable to swallow foods were prepared to control symptoms or finish at the same time as others [23, 26]. Sometimes, more palatable food would have been requested; less spicy, dry, or acidic [22, 23]. Alternatively, bringing their own supply of items such as custards, sauces, and gravies to modify meals made food more manageable in restaurants [31, 33]. To participate socially, on occasions, patients had to choose less appetising options [23].

At social gatherings, some patients would eat alone or requested family members to eat before them [36, 37]. Alternatively, some patients would eat in their own homes prior to meeting friends or family. This ensured they could eat their food but still participate and gain value from spending time with those in their social network [22, 37]. Others would choose to invite friends and family to their house instead of going out in order to take control of the food that was available [34].

##### Theme 2.2: Engaging with support

Whilst patients demonstrated a range of strategies in which they employed to cope with the physiological, psychological, and social aspects of eating, support from family and friends was paramount. In these circumstances, friends and family were generally described as supportive, helpful, and encouraging [22]. Some ways family members were able to support people included adapting meals, cooking alternative meals, and the alteration of eating patterns by the patient’s spouse [22, 30].

Following treatment some individuals reported feeling a sense of security when eating with family and close friends, as this alleviated stress and promoted confidence at mealtimes [22, 33]. For parents of young children, mealtimes were a positive experience as they were cooking, feeding or entertaining the children, which provided a sense of purpose and meaning [36].

There was little evidence of support received from HCPs to help promote patients’ experience of social eating. There were some indications of how HCPs could respond to the social eating challenges for people living with and beyond HNC. These included adopting holistic approaches providing education beyond the physical side effects of functional challenges and nutritional status of food items throughout the trajectory of recovery [22, 31, 34].

## Discussion

This is the first systematic review synthesising the social eating and drinking experiences for patients following treatment for HNC. The findings articulate an array of essential losses for patients following HNC, including a loss of confidence to eat socially, loss of taking part in social events, and loss of family togetherness. This review identified that patients were more likely to seek and receive support from someone within their close social network, rather than an HCP. The essential role of family during recovery and in cancer survivorship is highlighted. This body of literature primarily demonstrates the challenges associated with social eating and drinking after HNC treatment, with minimal findings on strategies to positively promote eating socially representing a gap in the current evidence-base.

Within the findings, it was reported that the social network with whom patients ate changed after treatment. Unfortunately, for some patients, mealtimes were no longer a social occasion and was viewed as a significant loss [44]. Eating was missed because of the taste and flavour of food; however, people chiefly mourned the loss of inclusion and belonging that food brings, including cultural and personal identity [18]. Challenges with social eating can inhibit the meaning of meals and restrict family togetherness within the home [44, 45]. There is a potential risk of reduced quality of life for patients with HNC who have challenges with eating and drinking. The functional challenges of HNC treatment and lack of social integration place people at risk of social isolation, loneliness, and poorer health-related quality of life [46, 47].

Wittmann et al. [48] described the interconnected biopsychosocial losses and successive feelings of grief in patients and partners after treatment for cancer. In viewing social eating and drinking challenges as a process of grief as opposed to a one-time event, it reframes the concept as an area that requires ongoing acknowledgement and potential continued support. Patients undergoing treatment for HNC may not be fully prepared or anticipate the ongoing functional challenges resulting from treatment [37]. For many patients, functional side effects can improve over time, however for some, it is slow, and for others, they never regain pre-treatment function [49, 50]. Social eating can continue to be a problem beyond 12 months post-treatment and a contributing factor to overall inferior quality of life [51]. Living with cancer as a chronic illness is a life-long process that requires adaption and change [52]. Some patients may come to the point of acceptance; however, additional research should examine the change of perception and experience of social eating across the trajectory from diagnosis to acute and long-term recovery.

An assessment tool developed by Burges-Watson et al. [18] provides a holistic framework of assessment for patients with altered eating challenges, recognising the biological causes, psychological consequences, and social impact. As there are a wide variety of activities associated with eating and drinking, HCPs must ensure eating and drinking needs of patients are explored beyond the physical domains. Patients reported a range of emotional reactions relating to social eating such as frustration, irritation, and anxiety. However, there is a paucity of information on how people coped with any emotional pain. Given the biopsychosocial challenges that accompany eating and drinking with others, a multi-disciplinary approach is required to ensure support extends beyond the mechanical and functional tasks of eating and drinking. Future research must consider how people cope beyond the physical and practical adjustments that they make, investigating the psychological, emotional, and relational domains.

Families are at the core of social eating and the key support providers for people with HNC [1]. Despite, sometimes feeling misunderstood, the assistance of family and friends was invaluable to overcoming social eating challenges and often the primary source for patients to seek support [46, 53]. As close relationships appear to be the most important means of support, it is essential to explore and understand the experience of family and close friends. Strategies for communal coping should be developed as both members of a relationship are involved with managing chronic illness [54]. Existing research by Patterson et al. [45] and Nund et al. (C) [55] explicitly investigate the experiences of family members of people with social eating challenges after treatment for HNC and conclude the potential burden of caring for someone with functional eating and drinking challenges. The need for feasible and acceptable family-based interventions has been previously indicated [56].

Given the wide-ranging impact of altered eating and drinking, it is imperative that studies investigate possible interventions that provide meaningful approaches to facilitate positive social participation in eating and drinking environments for patients and family members. Whittemore and Dixon [57] suggested that the key to managing a chronic illness is self-integration. Whilst coping, integration, and adjustment were demonstrated by participants in these findings, there was little detail on the process of acceptance or self-management. Recent work by Dunne et al. [21] provides evidence of incorporating self-management strategies into HNC survivorship programmes. Part of this process includes trial and error techniques [34], goal setting [52], and incorporation of self-management strategies [21]. As Dunne [21] reported that self-management works best in the context of individualised approaches. As people, cancers and treatments are different, patients may benefit from having interventions provided at different times, where short- and long-term interventions are used to complement each other [52].

Within the literature, there are no findings on the social experiences for people with HNC who require temporary or permanent nutrition via a tube, for example, a percutaneous endoscopic gastrostomy (PEG) or radiologically inserted gastrostomy (RIG). Further research is necessary for patients who are likely to have exacerbated challenges from specific treatments and have particular functional challenges such as laryngectomies, tracheostomies, and glossectomies.

## Limitations

No studies in this review had a specific aim to explore social eating experiences. Information on this topic was noted during the reporting of other topics, but the phenomenon of social eating and drinking was not explored in depth. The findings of this review did not differentiate the type or location of cancer or the treatment each person received. Further research is required to investigate the unique social eating challenges related to treatment modality or HNC subsite. Quality assessment was completed, but no study was rejected due to the quality of methodology. However, all the included papers had a score between 6 and 9 in the CASP tool, indicating that these were moderate to high-quality studies. The initial process of identifying literature was completed by the first author and the process checked by the research team. To enhance rigour, this process would have been completed independently by an additional researcher.

## Conclusion

The physical side effects of treatment for patients with HNC impact functional eating and drinking abilities in acute and long-term recovery. This affects both a person’s ability and confidence to eat and drink in front of others, thus leading to important losses in their life. These losses have emotional, relational, and cultural significance. Many people with HNC demonstrated resilience and overcame barriers by adapting and coping with eating and drinking socially, with support from family and friends being pivotal. Family- or couple-orientated resources should be developed to provide support to the person with HNC and their close family.

## Supplementary Information

ESM 1 (DOCX 58 kb)

## Data Availability

This is a systematic review of previously published data. Data has been referenced and attributed to its source within the review.
