# Supplementary material for: A qualitative systematic review of the social eating and drinking experiences of patients following treatment for head and neck cancer
Source: Support Care Cancer. 2021 Mar 1;29(9):4899–909. doi: 10.1007/s00520-021-06062-7 (PMC8295127; doi:10.1007/s00520-021-06062-7)
Supplement: Supplementary file 1 — (DOCX 58 kb) [file 520_2021_6062_MOESM2_ESM.docx]

| **Section/topic** | **#** | **Checklist item** | **Reported on page #** |
| --- | --- | --- | --- |
| **TITLE** | | |  |
| Title | 1 | Identify the report as a systematic review, meta-analysis, or both. | 1 |
| **ABSTRACT** | | |  |
| Structured summary | 2 | Provide a structured summary including, as applicable: background; objectives; data sources; study eligibility criteria, participants, and interventions; study appraisal and synthesis methods; results; limitations; conclusions and implications of key findings; systematic review registration number. | 3 |
| **INTRODUCTION** | | |  |
| Rationale | 3 | Describe the rationale for the review in the context of what is already known. | 4-5 |
| Objectives | 4 | Provide an explicit statement of questions being addressed with reference to participants, interventions, comparisons, outcomes, and study design (PICOS). | 5 |
| **METHODS** | | |  |
| Protocol and registration | 5 | Indicate if a review protocol exists, if and where it can be accessed (e.g., Web address), and, if available, provide registration information including registration number. | 6 |
| Eligibility criteria | 6 | Specify study characteristics (e.g., PICOS, length of follow-up) and report characteristics (e.g., years considered, language, publication status) used as criteria for eligibility, giving rationale. | 6 |
| Information sources | 7 | Describe all information sources (e.g., databases with dates of coverage, contact with study authors to identify additional studies) in the search and date last searched. | 6 |
| Search | 8 | Present full electronic search strategy for at least one database, including any limits used, such that it could be repeated. | 6, 9, 38-39 |
| Study selection | 9 | State the process for selecting studies (i.e., screening, eligibility, included in systematic review, and, if applicable, included in the meta-analysis). | 6-7 |
| Data collection process | 10 | Describe method of data extraction from reports (e.g., piloted forms, independently, in duplicate) and any processes for obtaining and confirming data from investigators. | 6-7 |
| Data items | 11 | List and define all variables for which data were sought (e.g., PICOS, funding sources) and any assumptions and simplifications made. | 6-7 |
| Risk of bias in individual studies | 12 | Describe methods used for assessing risk of bias of individual studies (including specification of whether this was done at the study or outcome level), and how this information is to be used in any data synthesis. | 8 |
| Summary measures | 13 | State the principal summary measures (e.g., risk ratio, difference in means). | 10 |
| Synthesis of results | 14 | Describe the methods of handling data and combining results of studies, if done, including measures of consistency (e.g., I^2^) for each meta-analysis. | 10 |

Page 1 of 2

| **Section/topic** | **#** | **Checklist item** | **Reported on page #** |
| --- | --- | --- | --- |
| Risk of bias across studies | 15 | Specify any assessment of risk of bias that may affect the cumulative evidence (e.g., publication bias, selective reporting within studies). | 8 |
| Additional analyses | 16 | Describe methods of additional analyses (e.g., sensitivity or subgroup analyses, meta-regression), if done, indicating which were pre-specified. | N/A |
| **RESULTS** | | |  |
| Study selection | 17 | Give numbers of studies screened, assessed for eligibility, and included in the review, with reasons for exclusions at each stage, ideally with a flow diagram. | 9-10 |
| Study characteristics | 18 | For each study, present characteristics for which data were extracted (e.g., study size, PICOS, follow-up period) and provide the citations. | 12-17 |
| Risk of bias within studies | 19 | Present data on risk of bias of each study and, if available, any outcome level assessment (see item 12). | 41-42 |
| Results of individual studies | 20 | For all outcomes considered (benefits or harms), present, for each study: (a) simple summary data for each intervention group (b) effect estimates and confidence intervals, ideally with a forest plot. | 12-17 |
| Synthesis of results | 21 | Present results of each meta-analysis done, including confidence intervals and measures of consistency. | 10-11,18-21 |
| Risk of bias across studies | 22 | Present results of any assessment of risk of bias across studies (see Item 15). | N/A |
| Additional analysis | 23 | Give results of additional analyses, if done (e.g., sensitivity or subgroup analyses, meta-regression [see Item 16]). | N/A |
| **DISCUSSION** | | |  |
| Summary of evidence | 24 | Summarize the main findings including the strength of evidence for each main outcome; consider their relevance to key groups (e.g., healthcare providers, users, and policy makers). | 22-25 |
| Limitations | 25 | Discuss limitations at study and outcome level (e.g., risk of bias), and at review-level (e.g., incomplete retrieval of identified research, reporting bias). | 25 |
| Conclusions | 26 | Provide a general interpretation of the results in the context of other evidence, and implications for future research. | 25 |
| **FUNDING** | | |  |
| Funding | 27 | Describe sources of funding for the systematic review and other support (e.g., supply of data); role of funders for the systematic review. | 26 |

**Pubmed** (1091) (17.2.20)

1) Head and neck cancer*

2) Head and neck neoplasm*

3) Head and neck tumour*

4) Head and neck tumor*

5) Oral cancer*

6) Oral neoplasm*

7) Oral tumour*

8) Oral tumor*

9) Mouth cancer*

10) Mouth neoplasm*

11) Mouth tumour*

12) Mouth tumor*

13) Laryn* cancer*

14) Laryn* neoplasm*

15) Laryn tumour*

16) Laryn tumor*

17) Pharyn* cancer*

18) Pharyn* neoplasm*

19) Pharyn* tumour*

20) Pharyn tumor*

21) 1 – 20 OR

22) eat*

23) drink*

24) food

25) swallow*

26) deglutition

27) dysphagia

28) 22 – 27 OR

29) social*

30) relation*

31) experience*

32) 29 – 31 OR

33) 21 AND 28 AND 32

**Scopus** (1437) (17.2.20)

1) Head and neck cancer*

2) Head and neck neoplasm*

3) Head and neck tumo?r*

4) Oral cancer*

5) Oral neoplasm*

6) Oral tumo?r*

7) Mouth cancer*

8) Mouth neoplasm*

9) Mouth tumo?r*

10) Laryn* cancer*

11) Laryn* neoplasm*

12) Laryn* tumo?r*

13) Pharyn* cancer*

14) Pharyn* neoplasm*

15) Pharyn* tumo?r*

16) 1 – 15 OR

17) eat*

18) drink*

19) food

20) swallow*

21) deglutition

22) dysphagia

23) 17 – 22 OR

24) social*

25) relation*

26) experience*

27) 24 – 26 OR

28) 16 AND 23 AND 27

**PsycINFO** (57) (17.2.20)

1) Head and neck cancer* - kw

2) Head and neck neoplasm* - kw

3) Head and neck tumo?r* - kw

4) Oral cancer* - kw

5) Oral neoplasm* - kw

6) Oral tumo?r* - kw

7) Mouth cancer* - kw

8) Mouth neoplasm* - kw

9) Mouth tumo?r* - kw

10) Laryn* cancer* - kw

11) Laryn* neoplasm* - kw

12) Laryn* tumo?r*

13) Pharyn* cancer* - kw

14) Pharyn* neoplasm* - kw

15) Pharyn* tumo?r - kw

16) 1 – 15 OR

17) eat* - kw

18) drink* - kw

19) food - kw

20) food - SH

21) swallow* - kw

22) swallowing - SH

23) deglutition - kw

24) dysphagia – SH

25) dysphagia - kw

26) 17 – 25 OR

27) social* - kw

28) relation* - kw

29) experience* - kw

30) 27 – 29 OR

31) 16 AND 26 AND 30

**CINAHL** (631) (17.2.20)

1) Head and neck cancer* - kw

2) Head and neck neoplasm* - kw

3) Head and neck tumo?r* - kw

4) Head and neck neoplasms – MH

5) Oral cancer* - kw

6) Oral neoplasm* - kw

7) Oral tumo?r - kw

8) Mouth cancer* - kw

9) Mouth neoplasm* - kw

10) Mouth tumo?r – kw

11) Mouth neoplasms – MH

12) Laryn* cancer* - kw

13) Laryn* neoplasm* - kw

14) Laryn* tumo?r* - kw

15) Laryngeal neoplasms – MH

16) Pharyn* cancer* - kw

17) Pharyn* neoplasm* - kw

18) Pharyn* tumo?r - kw

19) Pharyngeal neoplasms – MH

20) 1 – 19 OR

21) eat* - kw

22) eating - MH

23) drink* - kw

24) Food – kw

25) Food – MH

26) Swallow* - kw

27) Deglutition – MH

28) Dysphagia - kw

29) deglutition disorders - MH

30) Deglutition - kw

31) 21 – 30 OR

32) social* - kw

33) relation* - kw

34) experience* - kw

35) 31 – 34 OR

36) 20 AND 31 AND 35

**Web of Science** (1373) (17.2.20)

1) Head and neck cancer*

2) Head and neck neoplasm*

3) Head and neck tumo?r*

4) Oral cancer*

5) Oral neoplasm*

6) Oral tumo?r*

7) Mouth cancer*

8) Mouth neoplasm*

9) Mouth tumo?r*

10) Laryn* cancer*

11) Laryn* neoplasm*

12) Laryn* tumo?r*

13) Pharyn* cancer*

14) Pharyn* neoplasm*

15) Pharyn* tumo?r*

16) 1 – 15 OR

17) eat*

18) drink*

19) food

20) swallow*

21) deglutition

22) dysphagia

23) 17 – 22 OR

24) social*

25) relation*

26) experience*

27) 24 – 26 OR

28) 16 AND 23 AND 27

Key:

MH – Medical heading

SH – Subject heading

kw - keyword

**EMBASE** (2316) 17.2.20

1) Head and neck cancer - MH

2) Mouth tumor – MH

3) Pharynx cancer – MH

4) Head and neck cancer* - kw

5) Head and neck neoplasm* - kw

6) Head and neck tumo?r* - kw

7) Oral cancer* - kw

8) Oral neoplasm* - kw

9) Oral tumo?r* - kw

10) Mouth cancer* - kw

11) Mouth neoplasm* - kw

12) Mouth tumo?r - kw

13) Laryn* cancer* - kw

14) Laryn* neoplasm* - kw

15) Larynx* tumo?r* - kw

16) Pharyn* cancer* - kw

17) Pharyn* neoplasm* - kw

18) Pharyn* tumo?r*

19) 1 – 18 OR

20) eating – SH

21) dysphagia - SH

22) food – SH

23) Swallowing - SH

24) eat* - kw

25) drink*

26) dysphagia – kw

27) deglutition - kw

28) swallow* - kw

29) food - kw

30) 20 – 29 OR

31) experience - SH

32) social* - kw

33) relation* - kw

34) experience* - kw

35) 31 – 34 OR

36) 19 AND 30 AND 34

Systematic Review Screening Tool (Version 3)

**Review Question**: What are the social eating and drinking experiences for people living with and beyond head and neck cancer (HNC) and their family?

| **Population** | **Include**  Patients aged 18 or over | **Exclude**  Patients under age of 18 |
| --- | --- | --- |
| **Type of cancer** | **Include**  Head and neck cancer | **Exclude**  All other types of cancer  Data cannot be separated according to cancer type |
| **Treatment** | **Include**  Surgery, radiotherapy, chemotherapy or combination | **Exclude**  End-of-life |
| **Exposure** | **Include**  Eating and drinking difficulties following HNC treatment  Challenges with food and drink following HNC treatment | **Exclude**  Eating and drinking difficulties during HNC treatment |
| **Outcome** | **Include**  Social experience involving eating and drinking or food and drink  Social experience involving a place associated with food or drink | **Exclude**  Social experience not involving eating and drinking or food and drink |
| **Research type** | **Include**  Primary research | **Exclude**  Secondary research, systematic reviews, opinion articles, editorials, abstract only available |
| **Study design** | **Include**  Any | **Exclude**  Not excluded due to design |
| **Overall decision**  **with reason** | **INCLUDED** | **EXCLUDED** |

Reviewer name: Date:

Authors: Year:

Title: Journal:

Notes:

|  | 1. Was there a clear statement of the aims of the research? | 2. Is a qualitative methodology appropriate? | 3. Was the research design appropriate to address the aims of the research? | 4. Was the recruitment strategy appropriate to the aims of the research? | 5. Was the data collected in a way that addressed the research issue? | 6. Has the relationship between research and participants been adequately addressed? | 7. Have ethical issues been taken into consideration? | 8. Was the data analysis sufficiently rigorous? | 9. Is there a clear statement of findings? | 10. Overall score |
| --- | --- | --- | --- | --- | --- | --- | --- | --- | --- | --- |
| Alberda et al. (2017) | Yes | Yes | Yes | Yes | Yes | Yes | Yes | Yes | Yes | 9 |
| Burges-Watson et al (2018) | Yes | Yes | Yes | Yes | Yes | Can’t tell | Yes | Yes | Yes | 8 |
| Checklin et al. (2019) | Yes | Yes | Yes | Yes | Yes | Yes | Yes | Yes | Yes | 9 |
| Dooks et al. (2012) | Yes | Yes | Yes | Yes | Yes | No | Yes | Yes | Yes | 8 |
| Dunne et al. (2019) | Yes | Yes | Can’t tell | Yes | Yes | No | Yes | Yes | Yes | 7 |
| Einarsson et al. (2019) | Yes | Yes | Yes | Yes | Yes | No | Yes | Can’t tell | Yes | 7 |
| Ganzer et al. (2015) | Yes | Yes | Yes | Yes | Yes | No | Yes | Yes | Yes | 8 |
| Goswami & Gupta (2019) | No | Yes | Yes | Yes | Yes | No | Yes | Can’t tell | Yes | 6 |
| Jiang et al. (2017) | Yes | Yes | Yes | Yes | Yes | No | Yes | Can’t tell | Yes | 7 |
| McQuestion et al. (2011) | Yes | Yes | Yes | Yes | Yes | No | Can’t tell | Yes | Yes | 7 |
| Molassiotis and Rogers (2012) | Yes | Yes | Yes | Yes | Yes | No | Yes | Yes | Yes | 8 |
| Moore et al. (2014) | Yes | Yes | Yes | Yes | Yes | No | Yes | Yes | No | 7 |
| Mortensen & Paaske (2012) | Yes | Yes | Yes | Yes | Yes | No | Yes | Can’t tell | Yes | 7 |
|  | 1. Was there a clear statement of the aims of the research? | 2. Is a qualitative methodology appropriate? | 3. Was the research design appropriate to address the aims of the research? | 4. Was the recruitment strategy appropriate to the aims of the research? | 5. Was the data collected in a way that addressed the research issue? | 6. Has the relationship between research and participants been adequately addressed? | 7. Have ethical issues been taken into consideration? | 8. Was the data analysis sufficiently rigorous? | 9. Is there a clear statement of findings? | 10. Overall score |
| Nund et al. (A) (2014) | Yes | Yes | Yes | Yes | Yes | No | Yes | Yes | Yes | 8 |
| Nund et al. (B) (2014) | Yes | Yes | Yes | Yes | Yes | No | Yes | Yes | Yes | 8 |
| O’Brien et al. (2012) | Yes | Yes | Yes | Yes | Yes | No | Yes | Yes | Yes | 8 |
| Ottosson et al. (2013) | Yes | Yes | Yes | Yes | Yes | Yes | Yes | Yes | Yes | 9 |
| Parahoo et al. (2019) | Yes | Yes | Yes | Yes | Yes | No | Yes | Can’t tell | Yes | 7 |
| Pateman et al. (2015) | Yes | Yes | Yes | Yes | Yes | No | Yes | Can’t tell | Yes | 7 |
| Patterson et al. (2015) | Yes | Yes | Yes | Yes | Yes | No | Yes | Yes | Yes | 8 |
| Semple et al. (2019) | Yes | Yes | Yes | Yes | Yes | No | Yes | Can’t tell | Yes | 7 |
| Sterba et al. (2017) | Yes | Yes | Yes | Yes | Yes | No | Yes | Can’t tell | Can’t tell | 6 |
| Tong et al. (2011) | Yes | Yes | Yes | Yes | Yes | No | Yes | Yes | Yes | 8 |
| Zou et al. (2015) | Yes | Yes | Yes | Yes | Yes | Yes | Yes | Yes | Yes | 9 |
